# Oncolytic vaccinia virus and cancer immunotherapy

**DOI:** 10.3389/fimmu.2023.1324744

**Published:** 2024-01-12

**Authors:** Lihua Xu, Huihui Sun, Nicholas R. Lemoine, Yujing Xuan, Pengju Wang

**Affiliations:** ^1^ Sino-British Research Centre for Molecular Oncology, National Centre for International Research in Cell and Gene Therapy, State Key Laboratory of Esophageal Cancer Prevention & Treatment, School of Basic Medical Sciences, Academy of Medical Sciences, Zhengzhou University, Zhengzhou, China; ^2^ Centre for Biomarkers & Biotherapeutics, Barts Cancer Institute, Queen Mary University of London, London, United Kingdom

**Keywords:** oncolytic virotherapy, vaccinia virus, cancer immunotherapy, combination therapy, tumor microenvironment

## Abstract

Oncolytic virotherapy (OVT) is a promising form of cancer treatment that uses genetically engineered viruses to replicate within cancer cells and trigger anti-tumor immune response. In addition to killing cancer cells, oncolytic viruses can also remodel the tumor microenvironment and stimulate a long-term anti-tumor immune response. Despite achieving positive results in cellular and organismal studies, there are currently only a few approved oncolytic viruses for clinical use. Vaccinia virus (VACV) has emerged as a potential candidate due to its ability to infect a wide range of cancer cells. This review discusses the mechanisms, benefits, and clinical trials of oncolytic VACVs. The safety and efficacy of different viral backbones are explored, as well as the effects of oncolytic VACVs on the tumor microenvironment. The potential combination of oncolytic VACVs with immunotherapy or traditional therapies is also highlighted. The review concludes by addressing prospects and challenges in the field of oncolytic VACVs, with the aim of promoting further research and application in cancer therapy.

## 1 Introduction

Oncolytic virotherapy (OVT) is an emerging tumor treatment modality that is based on naturally or genetically engineered oncolytic viruses (OVs) to selectively lyse tumor cells while sparing healthy cells (1). The history of OVs include three stages: the discovery and application of wild-type viruses (before 1990), the research and development of genetically engineered viruses (1991–2000), and the insertion of therapeutic genes in viruses and synergistic therapies (21st century) (2). In 1991, Martuza et al. first reported a genetically engineered herpes simplex virus (HSV)-1 (dlsptk) for inhibiting glioma growth in nude mice, which accelerated the field of OVT (3). With deeper insight into the anti-tumor mechanisms, OVs have been deemed to not only lyse tumor cells selectively, but activate anti-tumor immunity and remodel the tumor microenvironment (TME), making them promising as oncologic therapeutic agents (4).

According to their genomic characteristics, OVs are divided into two categories, DNA viruses (e.g., adenovirus, vaccinia virus (VACV), herpesvirus) and RNA viruses [e.g., reovirus, measles virus, New Castle disease virus (NDV), vesicular stomatitis virus (VSV)], in which DNA oncolytic viruses are superior to other viruses due to their larger genome, genetic stability, and high replication ability (5). Larger genome allows for more flexibility for the expression of recombinant payloads. Most of the currently approved OVs that include Rigvir (enteric cytopathic human orphan virus approved in Latvia for melanoma in 2004), Oncorine (adenovirus approved in China for head and neck cancer in 2005), T-VEC (HSV approved in the United States for melanoma in 2015), and DELYTACT (HSV approved in Japan for glioblastoma in 2021) are DNA viruses. Besides the above approved OVs, many other candidate OVs show promising pre-clinical evidence of anti-tumor activity, and increasing clinical trials are currently underway to determine the efficacy of OVs in different cancer patients (6). However, the number of approved OVs and the applicable cancer types remain very rare in the clinic. Intratumor administration is one of the most important limiting factors. The presence of preexisting antiviral neutralizing antibodies or their development during viral therapy render repeat systemic treatments of OVs ineffective, limiting the application in some cancer types with metastases or unable to be administrated *in situ*. For instance, T-VEC is only approved in patient without visceral metastases. The development of novel and more potent oncolytic viruses is urgently needed.

As one kind of DNA virus, VACV is a large DNA prototypic poxvirus that replicates exclusively in the cytoplasm, and it is therefore fully nonintegrative. VACV has been used as a smallpox vaccine for many years with relatively low adverse reactions (7). Recent preclinical results and clinical data about different engineered oncolytic VACVs [e.g., Pexa-Vec (JX-594)] also show its potential for intravenous infusion and tumor therapy *via* highly and stably expressing many therapeutic genes (8). According to the phase 1b trial results of biweekly intravenous Pexa-Vec in colorectal cancer, no patients developed Pexa-Vec infusion-related reactions. The common adverse effects mainly include headache, nausea, anorexia, rash, and vomiting (9). Hence, VACVs are expected to be safe candidates for OVT.

This review outlines the characteristics, viral backbones and clinical trials of oncolytic VACVs. The anti-tumor mechanisms and effects on tumor microenvironment are also discussed. In addition, the potential combination treatments with immunotherapy or traditional therapies are highlighted. The prospects and challenges of oncolytic VACVs are also addressed to promote the further research and application in cancer treatment.

## 2 Characteristics and anti-tumor mechanisms of oncolytic VACVs

### 2.1 Characteristics of oncolytic VACVs

VACV is an enveloped double stranded DNA orthopoxvirus, in which the most widely used experimental strains include Lister, Western Reserve (WR), Wyeth strains, Copenhagen strains, Ankara (also known as MVA), and Tiantan stains etc (10). MVA and Tiantan stains are non-replicative strains of VACVs for cancer vaccine, while the other strains are replication competent viruses. During replication, VACVs produce four infectious forms which differ in their outer membranes: intracellular mature virion (IMV), the intracellular enveloped virion (IEV), the cell-associated enveloped virion (CEV) and the extracellular enveloped virion (EEV) (11). The IMV is the most abundant infectious form, responsible for spread between host, while the CEV and EEV are responsible for cell-to-cell transfer and long-range dissemination within the host organism, respectively (12, 13).

Compared with other virus vectors, VACVs have many advantages in OVT. 1) VACVs replicate exclusively in the cytoplasm, with no integration of viral DNA into the host genome, indicating its safety (14). 2) VACVs have a large genome (~190 kb), capable of inserting and stably expressing exogenous therapeutic genes of at least 25 kb in a single vector (15). 3) VACVs own natural tumor tropism, potential for systemic administration (16). 4) VACVs have a rapid and lytic replication cycle. The first viruses can be released from the cells within 8 h after infection, and the infected cells can be destroyed after 48-72 h of infection. 5) VACVs can replicate in hypoxic conditions (17). 6) Because of no limitation on receptors during entry, VACVs exhibit high infectivity not only in various host species but also in a large range of tissues, beneficial for preclinical researches (18, 19).

In addition, like other oncolytic viruses, oncolytic VACVs can exert anti-tumor effect *via* oncolysis and activation of anti-tumor immune responses. To date, several studies have shown that deletion of some endogenous genes enhances the oncolysis of oncolytic VACVs. The key VACV genes and corresponding oncolytic functions are listed in the **Table 1**. Deletion those genes can improve the antitumor efficacy through multiple ways such as increasing the tumor selectivity, safety and anti-tumor immune response.

**Table 1:** Oncolytic functions of key VACV genes.

Gene name	Corresponding protein function	Oncolytic function after gene deletion	PMID
TK	Key enzymes for DNA replication	Increase tumor selectivity and safety	10678358
VGF	Polypeptide with amino acid sequence homology to epidermal growth factor and transforming growth factor alpha	Attenuate virulence and increase safety	3339716, 30420785, 2739561
B18R	Bind and remove secreted type-I IFNs	Enable IFN-dependent tumor selectivity and increase safety	18162040
F1L	Bind and inhibit the NLR family member NLRP1 as an apoptosis inhibitor	Increase safety and oncolysis	23603272, 31428674
N1L	Inhibit apoptosis and NF-κB activity	Enhance CD8^+^ T-cell memory and natural killer cell response	22194685, 25382035, 32217766, 18931086
B2R	Viral cGAMP-specific nuclease	Enhance IRF3 phosphorylation and type I IFN expression, improving antitumor immune response	30728498, 37016144
E5R	cGAS inhibitor	Induce much higher levels of type I IFN, improving antitumor immune response	37217469, 37145142

Based on the above advantages, oncolytic VACV is an alternative virus vector for OVT.

### 2.2 Anti-tumor mechanisms of oncolytic VACVs

**Figure 1:**
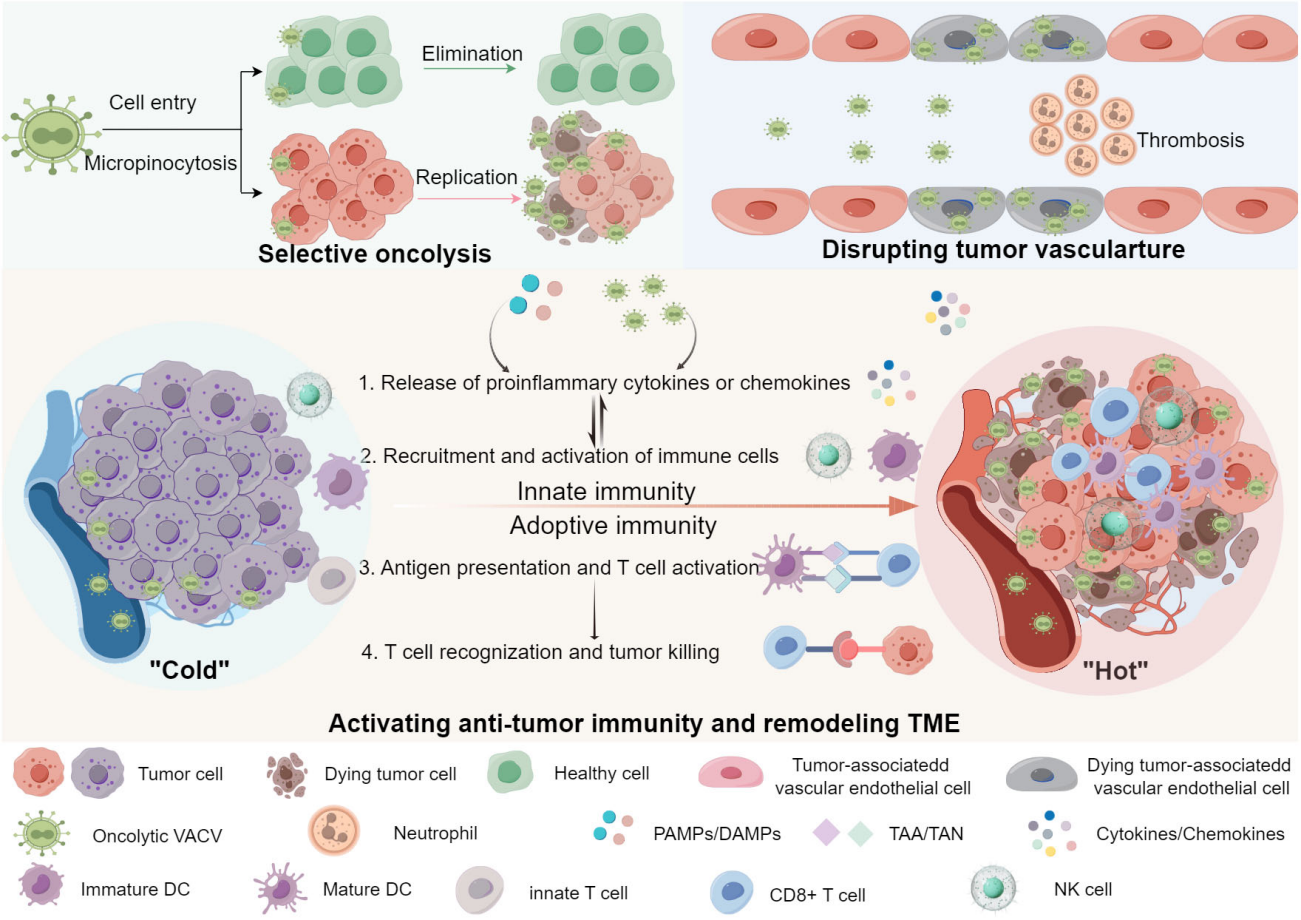
Anti-tumor mechanisms of oncolytic VACVs. Oncolytic VACVs can kill cancer cells *via* a variety of mechanisms. First, they directly infect, replicate and lyse tumor cells sparing normal cells. Released virions can infect neighbor tumor cells and so forth. Second, oncolytic VACVs can infect and lyse tumor associated vascular endothelial cells, meanwhile recruiting neutrophile cells and inducing thrombosis. Third, they can remodel the “cold” TME to “hot” by activating innate and adoptive anti-tumor immunity. The release of progeny viruses and PAMPs/DAMPs can promote the innate immune cells to produce proinflammatory cytokines and chemokines, which in turn lead to the recruitment and activation of more immune cells, thus innate immune responses are activated. With the activation of antigen presentation cells, DCs can present the released TAA/TAN to T-cells, enhancing the tumor recognition and killing ability of CD8^+^ T-cells, inducing a tumor-specific adoptive immune response. PAMPs, Pathogen-associated molecular patterns; DAMPs, Damage-associated molecular pattern; TAA, Tumor-associated antigens; TAN, Tumor-associated neoantigens; DC, Dendritic cell; NK cell, Natural killer cell. The figure is drawn by FigDraw.

Oncolytic VACVs primarily destroy tumor tissues *via* three mechanisms: direct oncolysis of tumor cells, disrupting tumor vasculature (tumor-associated endothelial cells lysis-mediated vascular collapse, neutrophils accumulation-mediated thrombosis) and activating anti-tumor immunity (**Figure 1**). In the following sections, we explore the detailed anti-tumor mechanisms of oncolytic VACVs.

#### 2.2.1 Selective self-replication and oncolysis in tumor cells

Unlike some viruses such as adenoviruses, VACVs have inherent tumor tropism. As one kind of invasive viruses, oncolytic VACVs infect host tumor cells in several steps (20). Firstly, oncolytic VACVs use distinct forms of macropinocytosis for host-cell entry, independent on receptors. A study from Helenius’s group demonstrated that mature virions (MVs) from both the WR strain and the International Health Department-J (IHD-J) strain entered host cells by macropinocytosis due to virion-exposed phosphatidylserine. However, different macropinocytic mechanisms were possible in the same cell line through subtle differences in the activating ligand. The results showed that MVs from the WR strain entered HeLa cells by activating transient plasma membrane blebbing, while MVs from the IHD-J strain induced rapid formation (and lengthening) of filopodia (19). Next, DNA and proteins for oncolytic VACVs replication are synthesized in the cytoplasm instead of the cell nucleus. Last, progeny of VACVs released from lysed tumor cells spread to surrounding uninfected tumor cells, leading to amplification of their oncolytic activity and inducing tumor cell death. The selective oncolysis of oncolytic VACVs mainly lies on the differences between tumor cells and normal cells. On the one hand, various tumor suppressor genes (e.g., p53, RAS, and PTEN) and antiviral signals (such as type I interferon (IFN) pathway) are significantly downregulated, which make it easier for oncolytic VACVs to survive and replicate in the tumor cell (21). On the other hand, most of oncolytic VACVs are constructed by deletion of some viral genes [e.g., thymidine kinase (TK)] that overexpressed in tumor cells (22). In the healthy cell, oncolytic VACVs cannot replicate due to the deletion of some viral genes that are essential for VACV replication. Once exposure of oncolytic VACV genome in the cytoplasm, cyclic GMP-AMP synthase (cGAS)/stimulator of interferon gene (STING) pathway is activated followed by the production of antiviral cytokines such as type I IFN, further inhibiting oncolytic VACV replication. Subsequently, the genome and proteins of oncolytic VACVs are degraded by DNA and protein-degrading enzymes.

#### 2.2.2 Disrupting tumor vasculature

In general, the peripheral region of a tumor contains enriched vasculature which is primarily composed of endothelial cells, and affects tumor growth and metastasis (23). Targeting the neovasculature is an alternative approach to eradication of tumor cells *via* starving and suffocating tumors. For the first time, Kirn and coworkers found that the oncolytic VACV (JX-594) could induce cytokines and chemokines-mediated neutrophils accumulation in blood vessels, leading to intravascular thrombosis (24). Soon afterwards, they revealed that JX-594, with deletion of TK genes, was able to specifically target and infect tumor-associated endothelial cells and efficiently replicate due to increased vascular endothelial growth factors (VEGF) signaling-mediated TK overexpression, contributing to vascular collapse (25). After intravenous injection of oncolytic VACVs, tumor perfusion in patient biopsy decreased from magnetic resonance imaging results, and no clinical signs of damage to normal vasculature were observed. Another research from Bell’s group revealed that VEGF/VEGFR2 signaling in remodeling vessels could also sensitize the tumor vasculature to oncolytic VACVs infection by PRD1-BF1/Blimp1-mediated antiviral immune suppression (26). However, Santry et al. considered that despite many potential benefits by oncolytic VACV-mediated tumor vascular collapse such as leukomonocyte recruitment, shutting off blood vessels may also limit oncolytic VACVs spread and obstruct the delivery of subsequent therapeutic agents, and the entry of immunological effector cells (27).

#### 2.2.3 Activating anti-tumor immune response and remodeling TME

Early the anti-tumor mechanism of OVT was recognized as direct oncolysis of OVs. With the development of tumor immunotherapy, the impact of OVT on the immune response to tumors has become widely studied. It is widely acknowledged that oncolytic VACVs can activate innate and adaptive immune systems in tumors, and further remodel tumor immunosuppressive microenvironment.

Generally, after infection by oncolytic VACVs, pathogen-associated molecular patterns (PAMPs) and progeny viruses are released and sensed by pattern recognition receptors (PRRs) on innate immune cells. Subsequently, chemokines (e.g., CCL3, CCL5, CXCL8, CXCL9) and proinflammatory cytokines (e.g., type I IFNs, interleukin (IL)-12, GM-CSF, TNF-α) are released to recruit and activate more innate immune cells such as macrophages, neutrophils, dentritic cells (DCs) and T-cells in the TME to eliminate the infected tumor cells, which can further stimulate the production of proinflammatory cytokines and chemokines to amplify the initial innate response (28).

In addition, accumulating evidences suggest that oncolytic VACVs induce immunogenic cell death (ICD) in infected cancer cells with the release of damage-associated molecular pattern molecules (DAMPs), such as the “danger” signals high mobility group box 1 (HMGB1), the “find me” signal adenosine triphosphate (ATP) and the “eat me” signal calreticulin (CRT) (29). The DAMPs can activate the immature DC (iDC) into the mature DC (mDC). mDCs are the main antigen presentation cells that present the released tumor-associated antigens (TAAs), and tumor-associated neoantigens (TANs) after oncolysis to T-cells, resulting in activation, proliferation and differentiation of T-cells (30). Effector T-cells exert specific tumor killing, meanwhile memory T-cells are responsible for long-term and distant anti-tumor effect. Thus, adaptive immune responses to oncolytic VACV-infected tumor cells are activated.

It can be seen from the clinical data reported by Samson et al. that JX-594 infusion contributed to a significant increase of IFN and neutralizing antibodies in plasma for viral clearance. Meanwhile, many other proinflammatory and chemokines such as IL12, CXCL10, CXCL2 also increased, leading to the activation of natural killer (NK) cells, and the recruitment and activation of T-cells and DCs (31).

As stated above, oncolytic VACV acts as a bait in the tumor to increase the number of immune cells and proinflammatory cytokines and chemokines in the TME, resulting in the amplification of the anti-tumor immune response and remodeling immunologically “cold” tumors into “hot” tumors. However, immune cells in the TME may be detrimental to effectiveness of OVT by eliminating virally infected cells. Antiviral immunity is a double-edged sword that needs to be well-balanced in tumor therapy (32). It is reflected not only in the tumor tissues, but in the blood circulation when intravenously administrating reovirus or coxsackievirus according to the study from Berkeley et al. (33). They found that neutralizing antibodies had an actual beneficial effect on OVT by forming virus/neutralizing antibody complexes which can be internalized by monocytes and delivered to tumor sites in spite of viral clearance. This finding is very exciting and beneficial for the understanding and application of OVT in future. However, whether this phenomenon exist in the oncolytic VACVs treatment still needs to be explored.

## 3 Construction of oncolytic VACV backbones

Compared with non-replicative VACVs, replication competent VACVs retain their ability to lyse tumor cells and spread through tumor tissues while the lower safety and efficacy limited the application. With the ever-increasing knowledge in the fields of molecular virology and cancer cell biology, engineered oncolytic VACVs can be obtained through the DNA recombinant technology (e.g., CRISPR-Cas9 system) (34). The multiple safe and efficacious tumor-targeted oncolytic VACV backbones have been developed in recent years *via* genome editing. Based on the function of the deletion genes, they are mainly classified into three types: viral replication-related, viral dissemination-related and antiviral immune evasion-related. The functions of some VACV genes are listed in the **Table 2**.

**Table 2:** Functions of some VACV genes^a^.

Classification	Gene name	Function	Reference
Viral replication-related	RR	Key enzymes for DNA replication	(35)
Viral dissemination-related	F13L	EEV and CEV generation and plaque formation	(36)
Anti-tumor immune evasion-related	F14L	Disrupt the binding of p65 to its co-activator CBP and reduce acetylation of p65-K310, inhibiting NF-κB	(37)
	B14R	Inhibit IκB kinase β and NF-κB activation	(38, 39)
	A49R	Inhibit NF-κB activation by binding to β-TrCP	(40)
	N2L	Inhibit activation of IFNβ promoter by inhibiting IRF3 activation	(41)
	K7R	Inhibit NF-κB and IRF activation	(42)
	B18R	Bind and remove secreted type-I IFNs, including IFN-β	(43)
	C21L	Inhibit complements activity	(44)
Multifunctional	A56R	Reduce superinfection and prevent cell–cell fusion of infected cells by forming A56-K2 complex, protect infected cells from complement attack by forming A56-VCP complex, and have haemagglutination activity	(45–47)

a The functions of omitted genes such as TK, VGF, B2R et al. were listed in **Table 1**.

Targeting the overexpressed proteins [e.g., TK and ribonucleotide reductase (RR)] in tumor cells that are necessary for viral replication is the common method to increase the safety of oncolytic VACVs (22, 35). JX-594 is a typical TK-deleted oncolytic VACV with confirmed safety in phase I clinical trials (e.g., NCT02977156 and NCT01394939). Double deletion of TK and RR also showed enhanced safety and selective replication in the preclinical studies (48).

Deletion of viral virulence genes can further enhance the safety of oncolytic VACVs. VACV virulence is related to viral replication, dissemination, and antiviral immune evasion. Viral growth factors (VGF) is an EGF homologue that activates EGFR-Ras pathway to promote cell proliferation. Studies have shown that deletion of VGF contributes to low replication and virulence (49, 50). Double deletion of TK and VGF led to significantly attenuated virulence in resting cells *in vitro* and tumor-specific replication *in vivo* (51). As mentioned previously, EEV and CEV forms of VACVs are responsible for viral dissemination *in vivo*. Deletion of the vaccinia virus F13L gene results in a highly attenuated virus that is defective in EEV and CEV generation and plaque formation (36).

Antagonizing antiviral immunity is an important way for oncolytic VACVs to potentiate virulence. cGAS/STING pathway plays a central role in immune defense against tumors and viral infections, where TANK-binding kinase 1 (TBK1) recruitment to STING further activates both NF-κB and interferon regulatory factor 3 (IRF3) (52). cGAS is a DNA sensor for host defense against VACV infection. Vaccinia B2R gene was recently discovered to encode a cytosolic cGAMP nuclease that obstruct the cGAS signal. Vaccinia E5 is another virulence factor that inhibits cGAS by abolishing cGAMP production. Studies showed that deletion of B2R or E5R gene made the virulence of VACVs attenuated and anti-tumor immunity enhanced (53, 54).

In addition, inhibition of NF-κB signaling and/or IFR signaling cannot only reduce viral virulence, but enhance anti-tumor immunity. F14 is a selective NF-κB inhibitor in the nucleus by disrupting the binding of p65 to its co-activator CBP and reducing acetylation of p65-K310 and a VACV strain lacking F14 has reduced virulence in a mouse model (37). There are many other proteins that show antagonism of NF-κB activation with different action sites. For instance, B14 interacts directly with IκB kinase β to inhibit its activation, and A49 inhibits NF-κB activation by binding to β-TrCP, contributing to virus virulence (38–40). Additionally, N2 is a nuclear virulence factor that inhibits activation of IFNβ promoter by inhibiting IRF3 activation, deletion of which reduced virulence of VACVs (41). Interestingly, some proteins (e.g., N1 and K7) have dual functions of inhibiting NF-κB and IRF activation (42, 55). Studies proved that deletion of the N1L gene reduced virulence and inhibited VACV replication, meanwhile it improved the generation of immediate and long-term memory CD8^+^ T-cell responses and induced a stronger NK cell response to infection (56, 57). Moreover, deletion of B8R or B18R that directly binds to several species of IFN and neutralizes the antiviral activity also makes oncolytic VACV attenuated for mice (43, 58).

The activation of complement system is another key innate immune defense against viral infection through classical and alternative pathways. Isaacs and coworkers demonstrated that VACV complement-control protein (VCP) could prevent antibody-dependent complement-enhanced neutralization of infectivity and contribute to virulence, as VCP gene (C21L) knockout viruses were attenuated in an intradermal rabbit model (44).

Notably, one gene may be involved in complex biological processes. For example, the VACV A56 protein with haemagglutination activity is able to bind two other viral proteins, a serine protease inhibitor (K2) and VCP, and express them at the surface of the infected cell. The A56–K2 complex binds to the entry–fusion machinery of VACV; reducing superinfection and preventing cell–cell fusion of infected cells, while the A56–VCP complex protects infected cells from complement attack (45, 46). However, the deletion of A56R did not attenuate VACVs in some cases according to the summary from DeHaven and coworkers (47). Given that A56R gene is non-essential for viral replication, it still can be used as a region of VACV genome suitable for exogenous gene insertion. GL-ONC1 is a typical VACV that inserts β-glucuronidase into the A56R loci of VACV genome. The increasing understanding of the multiple functions of VACV genes is conducive to optimize the VACV backbones.

## 4 Oncolytic VACV-based immune-related combination therapies

**Figure 2:**
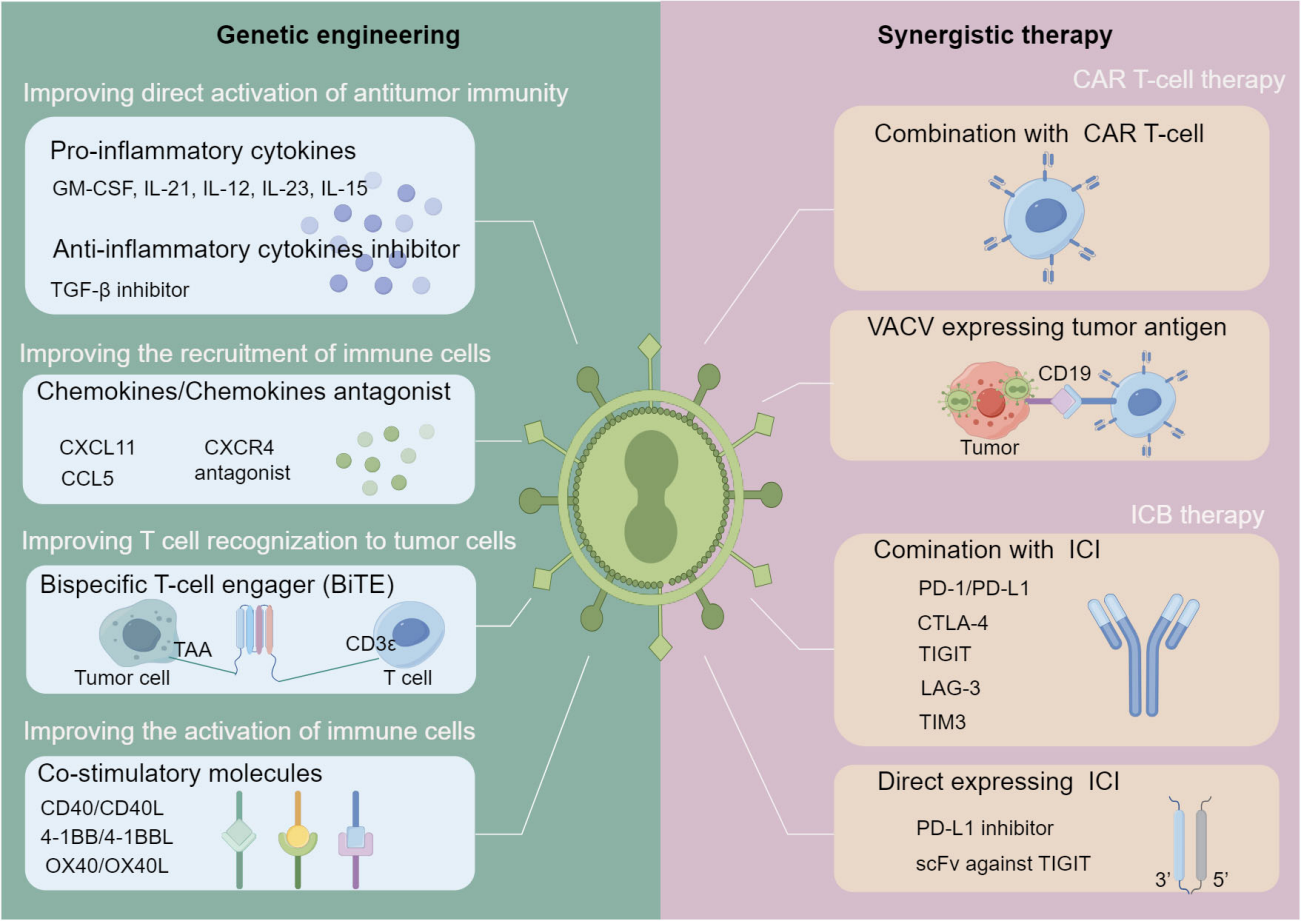
Strategies for oncolytic VACV-based immune-related combination therapies. Strategies are divided into two main categories: genetic engineering of oncolytic VACVs and combination with other immunotherapies. In terms of genome engineering (left), various immunostimulatory genes can be inserted into the genome of oncolytic VACVs, including those can activate the anti-tumor immunity directly, improve the T-cells recognition to cancer cells, recruit and activate immune cells. In terms of synergistic immunotherapies (right), oncolytic VACVs can be combined with CAR T-cell or ICI molecules directly or express CAR or ICI to enhance the synergistic therapies. oncolytic VACV, Oncolytic vaccinia virus; TAA, Tumor associated antigen; BiTE, Bispecific T-cell engager; CAR, Chimeric antigen receptor; ICI, Immune checkpoint inhibitor; scFv, Single chain variable fragment. The figure is drawn by FigDraw.

OVT is an effective form of immunotherapy that has been used to treat cancer. As previously mentioned, OVs can selectively kill tumor cells, activate anti-tumor immunity and remodel the “cold” TME to “hot”. However, OV-based monotherapy has restricted ability to activate anti-tumor immunity, given the potential antiviral machinery induced by activation of the IFN signaling pathway and the highly variable heterogeneity of malignant cells. In this section, strategies augmenting anti-tumor immune responses *via* synergistic therapies are summarized (**Figure 2**).

### 4.1 Oncolytic VACVs encoding immunostimulatory genes

With the ever-increasing knowledge of immune regulation mechanisms in tumor tissues, some immunostimulatory genes have been inserted into the genome of VACVs to amplify the anti-tumor immunity of oncolytic VACVs. Anti-tumor immunostimulatory factors include cytokines, chemokines, co-stimulatory factors and so on. Inflammatory cytokines are soluble proteins with the function of regulating innate and adoptive immunity, including pro-inflammatory and anti-inflammatory cytokines.

Arming pro-inflammatory cytokines is a usual way to enhance OVT by recruiting, activating immune cells and inhibiting immunosuppressive cells. GM-CSF is one of the most widely used cytokines that improve OVT. It recruits both DCs and NK cells to promote the maturation of DCs, which in turn, activates anti-tumor immunity (59). T-VEC, a GM-CSF armed HSV, has been approved in the United States for melanoma in 2015. JX-594, a genetically modified VACV that inserts the GM-CSF gene also has shown promising anti-tumor ability in clinical trials. IL-21 arming potentiated the anti-tumor activity of oncolytic VACVs by increasing effector CD8^+^ T-cell populations (60, 61). Some pro-inflammatory cytokines (e.g., IL-12, IL-23, IL-15) armed oncolytic VACVs showed stronger anti-tumor immune response by enhancing T-cell and NK cell activation and cytotoxicity in addition to increasing the production of IFN-γ (62–64). Other cytokines that have been used to engineer oncolytic viruses such as adenovirus and HSV may also enhance the anti-tumor effect of oncolytic VACVs through rational design (65). What is noteworthy is that assessment of actual exposure to potential payloads and management of safety issues that may arise from these payloads need to be considered when engineering oncolytic VACVs.

Owing to the important role of anti-inflammatory cytokines (e.g., TGF−β, IL-10) in host immune response, they are also designed to improve the therapeutic efficacy of OVT. Delgoffe and coworkers found that oncolytic VACV-delivered TGFβ inhibitor could overcome the immunosuppressive tumor microenvironment by blocking the immunosuppressive function of TGF−β, and increasing the sensitivity to INF-γ (66). The IL-10 is a recognized immunosuppressive cytokine that inhibits the production of pro-inflammatory cytokines such as IL-12 and INF-γ (67). Hickman and coworkers demonstrated that locally produced IL-10 after VACVs infection limited VACVs replication and dissemination (68). Another study from Wang and coworkers revealed IL-10 could also enhance the anti-tumor efficacy of oncolytic VACVs in pancreatic cancer by dampening antiviral immune response, and prolonging viral persistence in tumors (69).

Chemokines are secreted chemotactic cytokines that attract immune cells into tumor lesions and mediate anti-tumor immune effects. Bartlett’s group demonstrated that oncolytic VACVs encoding CCL5 or CXCL11 elicited potent anti-tumor immunity and enhanced therapeutic efficacy by attracting activated immune cells such as T (Th1) and NK cells (70, 71). Generally, chemokine receptors (e.g., CXCR4) help activate the immune system, while the regulation imbalance of signaling pathway promote the tumor growth and metastasis. CXCR4 antagonist armed oncolytic VACVs showed the ability of TME modulation such as inhibiting intratumoral accumulation of immunosuppressive myeloid-derived suppressor cells (MDSCs) and regulatory T-cells (Tregs) (72).

In addition, oncolytic viruses can be modified to express co-stimulatory molecules of T-cell, thereby enhancing T-cell-mediated anti-tumor immunity. ONCOLYTIC VACVs expressing co-stimulatory molecules (e.g., CD40L, 4-1BBL, OX40L) boosted anti-tumor immune responses by activating antigen presentation cells and T lymphocytes, and reprogramming Treg (73–75). Bispecific T-cell engager (BiTE) further improved the anti-tumor activity of oncolytic VACVs by expressing tumor specific antigen in tumor cells and redirecting T-cells to the tumor (76).

Apart from the immune stimulators, some genes associated with other signal pathways are also found to activate anti-tumor immunity. For example, expression of DNA-dependent activator of IFN-regulatory factors (DAI, a cytosolic dsDNA sensor) by an oncolytic VACV boosted innate immune activation and enhanced anti-tumor immunity (77). Oncolytic VACVs expressing White-Spotted Charr Lectin not only induced type I IFN production to elicit anti-tumor activity, but also inhibited IFN-induced ISG production, helping oncolytic VACVs escape elimination (78).

Taken together, oncolytic VACVs encoding genes that induce anti-tumor immune responses have the potential to promote stronger anti-tumor effect.

### 4.2 Oncolytic VACVs and chimeric antigen receptor (CAR) T-cell therapy

CAR T-cell therapy is a kind of adoptive immunotherapy aimed at augmenting the anti-tumor immune responses of T-cell. In CAR T-cell therapy, one or more CARs are expressed for both antigen-binding and T-cell activating, bypassing the antigen processing and presentation (79). Currently, two products targeting CD19-CAR (Tisagenlecleucel and Axicabtagene Ciloleucel) have been approved to treat hematologic malignancies, and many other CARs are being developed for solid tumors (80). However, the application of CAR T-cells in solid tumors faces several challenges, such as the reduced persistence and expansion of CAR T-cells in immunosuppressive TME and the antigen loss/escape (81, 82).

It is now recognized that oncolytic VACVs can activate the anti-tumor immune response and remodel the “cold” TME to “hot”. The enhanced type I IFN signature and the released DAMPs and cytokines in the TME after oncolysis of oncolytic VACVs can further support the priming, proliferation, clonal expansion, effector function and/or memory formation of CD8^+^ T-cells, enabling the anti-tumor effect of CAR T-cells (83). For example, the combination of HER-2-CAR T-cell and oncolytic VACV with deletion of TK and VGF showed a significant enhancement of tumor killing in the murine breast cancer (D2F2) cell line (84).

To enhance the tumor targeting of CAR T-cells, Priceman’s group designed an oncolytic VACV (OV19t) encoding a non-signaling, truncated human CD19 (CD19t) protein in which highly expressed CD19t on the surface of tumor cells worked as a specific tumor antigen to enable the tumor targeting of CD19-CAR T-cells (85). The combination therapy of OV19t/oncolytic viruses encoding the murine CD19t (OVm19t) protein and CD19-CAR T-cells showed durable and effective anti-tumor effects in MDA-MB-468/U251T bearing human tumor xenograft models and a MC38 bearing immunocompetent murine syngeneic tumor models. Another study from Aalipour et al. also demonstrated that the combination therapy of CD19 encoding oncolytic VACVs with CD19-CAR T-cells significantly reduced tumor growth and improved median survival achieved the similar results in an immunocompetent model of B16 melanoma (86). In addition, oncolytic VACVs encoding CXCL11 can further enhance the anti-tumor effect of mesothelin-CAR T-cell therapy *via* CXCL11-mediated recruitment of T-cells (87).

Taken together, oncolytic VACVs can boost the CAR T-cell therapy by the expression of tumor antigen or immunostimulatory factors and activation of anti-tumor immune responses themselves.

### 4.3 Oncolytic VACVs and immune checkpoint blockade therapy(ICB)

ICB therapy is an emerging immunotherapy aiming at blocking immunosuppressive tumor signals and restoring anti-tumor immune responses by targeting checkpoint receptors or ligands such as PD-1/PD-L1, CTLA-4, TIGIT, LAG-3, TIM3, among which PD-1/PD-L1 is the most common research object. To date, several immune checkpoint inhibitors (ICIs) have been approved for tumor treatment, including PD-1 inhibitors (nivolumab, pembrolizumab, cemiplimab), PD-L1 inhibitors (avelumab, durvalumab, atezolizumab), and CTLA-4 inhibitors (ipilimumab). However, the efficiency of ICIs is restricted by the immunosuppressive TME and low expression of immune checkpoint molecules.

Oncolytic VACVs cannot only recruit immune cells into immunodeficient tumors and remodel the “cold” TME to “hot”, but increase the expression of ICIs such as PD-L1, CTLA-4 or TIGIT inhibitors, sensitizing tumors to ICB therapy. The combination of CXCL11 encoded oncolytic VACV and PD-L1 blockade synergistically enhanced the therapeutic efficacy by increasing T-cell infiltration into the tumor and upregulating the expression of PD-L1 (88). The manganese superoxide dismutase (MnSOD) or immunostimulatory cytokines (such as IL-21, IL-15) expressing oncolytic VACVs could also improve PD-1/PD-L1 inhibition outcome by remodeling the suppressive tumor microenvironment (89–91). In addition, oncolytic VACVs have been engineered to directly express ICIs. For instance, oncolytic VACVs encoding a single chain variable fragment against TIGIT induced effective anti-tumor immunity and achieved a profound remodeling of the inhibitory tumor microenvironment from a “cold” state to a “hot” state synergizing with PD-1 or LAG-3 blockade (92). Oncolytic VACVs co-expressing ICIs and immune stimulatory cytokines exhibited synergistic anti-tumor ability. Oncolytic VACVs expressing PD-L1 inhibitors and GM-CSF activated tumor neoantigen-specific T-cell responses by synergistic action of VACV replication, GM-CSF stimulation, and PD-L1 inhibition on tumor cells and immune cells (93). Oncolytic VACVs expressing CTLA-4 inhibitors and GM-CSF elicited robust systemic CD8^+^ T-cell-dependent anti-tumor immunity and long-lasting anti-tumor immunity by expanding peripheral effector CD8^+^ T-cells and reducing Tregs and exhausted CD8^+^ T-cells (94).

Despite potential synergistic effect of oncolytic VACVs and ICIs, it is worth noting that ICIs may stimulate OVs clearance by activating host immune response. Thus, the optimal administration timing should be considered. Nguyen and coworkers concluded that OV lead-in → anti-PD-1 or OV lead-in → concurrent therapy could be the treatment option for tumor regression and eradication according to PubMed literature search results (95).

## 5 Other oncolytic VACV-based combination therapies

### 5.1 Oncolytic VACVs and radiotherapy (RT)

Radiotherapy (RT) is a type of conventional treatment for cancer by inducing DNA damage and apoptosis. With continuous exploration, combination of RT and oncolytic VACVs are recognized as a potent treatment modality for cancer. Focally irradiation in tumor tissues can facilitate the replication of systemically delivered oncolytic VACVs (96). In turn, VACVs encoding the sodium iodide symporter (NIS) can transfer radioactive iodine into virus-infected cancer cells, contributing to enhanced anti-tumor effects of RT in pancreatic cancer and breast cancer (97). Inspired by the theory of peptide-receptor radiotherapy (PRRT), and the ability of selected replication and exogenous gene expression of oncolytic VACVs, McCart and coworkers found that VACVs encoding somatostatin receptor (SSTR) led to virus-directed tumor-specific accumulation of radiopeptides, enabling the imaging and improved treatment of intraperitoneal CRC tumors using ^177^Lu-DOTATOC (98). In addition, studies have shown that radiation combined with oncolytic VACVs displayed considerably superior anti-tumor efficacy in several tumor models such as glioblastoma and pancreatic cancer (99, 100). For the synergistic mechanisms, a study from Chen et al. showed that RT combined with oncolytic VACVs could trigger tumor cell necroptosis and modify macrophages through the release of DAMPs, generating potent anti-tumor immunity and enhanced anti-tumor efficacy (101). Another study from Kyula et al. illustrated that synergistic cytotoxicity of radiation and oncolytic Lister strain VACV in V600D/E BRAF mutant melanoma depended on JNK and TNF-α signaling (102). Beside preclinical studies, phase II study about the combination of oncolytic VACV (GL-ONC1) with radiation and chemotherapy is warranted after phase I study in patients with locoregionally advanced head and neck carcinoma (103). All above studies proved the potential of the combination of RT and oncolytic VACVs.

### 5.2 Oncolytic VACVs and chemotherapy

Chemotherapy is the most widely used approach for tumor therapy due to the efficiency and broad spectrum. Chemotherapeutic drugs are cytotoxic agents that act primarily by inhibiting DNA replication or disrupting microtubule structures (104). Some of these cytotoxic agents have been extensively studied in combination with oncolytic VACVs and achieved many positive results. Yu and coworkers found that the anti-tumor efficacy of oncolytic VACV (GLV-1h68) was enhanced by cisplatin or gemcitabine with several potential mechanisms such as the changes in apoptosis, nucleotide pools and DNA repair pathways (105). As seen from the results of a phase II non-randomized clinical trial (NCT05281471) in patients with platinum-resistant or platinum-refractory ovarian cancer (PRROC), oncolytic VACV (Olvi-Vec) in combination with platinum-based chemotherapy demonstrated a favorable objective response rate (ORR) and progression-free survival (PFS) with a manageable safety profile in patients, and that patients are being recruited for phase III trial (NCT05281471) (106, 107). Cyclophosphamide (CPA) enhanced the replication of oncolytic VACVs by transiently suppressing the anti-viral immune response, while another study revealed that the enhanced anti-tumor efficacy of combining oncolytic VACV (GLV-1h68) with CPA was due to an effect on the vasculature rather than an immunosuppressive action of CPA (108, 109). In addition, Thorne’s group for the first time revealed that oncolytic VACVs could further sensitize tumor cells to chemotherapy such as anti-microtubule agent paclitaxel by inducing the release of several cytokines including type I IFN and HMGB1 (110). Some other chemotherapeutic drugs might boost OVT by synergistically activating ICD-mediated anti-tumor immunity such as doxorubicin (111).

Arming VACVs with a prodrug-activator gene is another approach to augment synergistic anti-tumor effects of OVT and chemotherapy with less systemic toxicity. Seubert and coworker demonstrated that GLV-1h68, an engineered oncolytic VACVs encoding β-galactosidase, exhibited enhanced oncolysis and tumor shrinkage with a β-galactosidase-activatable prodrug (112). GLV-1h94 encoding supercytosine deaminase (SCD) increased the cell specific-sensitivity of chemotherapeutic compound 5-fluorouracil (5-FU) by converting the prodrug 5-fluorocytosine (5-FC) to 5-FU only in the oncolytic VACV infected tumor cells (113). The addition of 5-FC made 85% of the cell lines highly sensitive to the combination treatment, none of which tested exhibited a “highly resistant” pattern. Nevertheless, the converted 5-FU reduced the replication of GLV-1h94 in tumor cells. The balance between cell line-specific susceptibility to GLV-1h94-induced oncolysis and 5-FU sensitivity should be taken into consideration.

### 5.3 Oncolytic VACVs and molecular targeted therapy

Different from chemotherapy, molecular targeted therapy, also known as “bio-missile”, is aimed at precisely killing tumor cells by targeted selection of blockers for certain key molecules that are overexpressed in tumor cells but not in normal cells. Given the lower toxicity, molecular targeted therapy has become a promising approach for tumor treatment.

Studies have shown that molecular targeted therapy can amplify the anti-tumor efficacy of oncolytic VACVs through several mechanisms such as evading antiviral immunity and increasing anti-tumor immunity. Inhibition of MEK-ERK pathway enhanced oncolytic VACVs accumulation in doxorubicin-resistant ovarian cancer by abrogating cytosolic DNA sensing and viral defense (114). Transient inhibition of PI3Kδ or COX-2 enabled repeated administration of oncolytic VACVs *via* inhibiting the monocyte uptake of VACVs or decreasing the production of neutralizing antibodies against oncolytic VACVs, with no effect on virus replication, respectively, enhancing the anti-tumor efficacy of IV-delivered oncolytic VACVs (115, 116). Histone deacetylase inhibitors (HDIs) and STAT3 inhibitors were found to enhance the spread and replication of oncolytic VACVs selectively and effectively in tumor cells by dampening innate antiviral immune response (117). What’s more, addition of a multitargeted receptor tyrosine kinase inhibitor sunitinib also amplified the anti-tumor effects of JX-594, by improving anti-tumor immune responses such as increasing CD8^+^ T-cell recruitment and decreasing Tregs and MDSCs (118). It is worth mentioning that the administration sequence should be considered because some drugs influence viral replication. For example, if given simultaneously *in vitro*, sorafenib could inhibit JX-594 replication (119). Hence, in the phase III trial of JX-594, sorafenib was administrated after JX-594 to avoid its antiviral effect. Oncolytic VACVs encoding GLAF-1 exhibited significantly enhanced therapeutic efficacy by directing against VEGF (120).

In a word, the combination of molecular targeted therapy and oncolytic VACVs will be a promising new anticancer therapy. More available molecules for tumor targeting and corresponding drugs need to be developed to enhance OVT.

## 6 Clinical trials with oncolytic VACVs

As the exciting anti-tumor research results increase, some potential engineered Oncolytic VACVs are warranted for clinical trials. The current clinical trials about replication-competent oncolytic VACVs are found from the website of https://clinicaltrials.gov, and listed in the **Table 3**. TK gene deletion is the most common way to increase the selectivity of oncolytic VACVs and different therapeutic genes are inserted for better anti-tumor effect. Pexa-Vec and GL-ONC1 are the only two oncolytic VACVs that are already in phase III clinical trials, indicating the safety and efficacy in some cancer patients. Unfortunately, the phase III trial of Pexa-Vec for hepatocellular carcinoma was declared a failure because the interim analysis of the study showed that it was unlikely to prolong the overall survival of patients with Pexa-Vec treatment. Perhaps other combination therapies such as ICB with OVT will exert promising anti-tumor effects.

**Table 3:** Current clinical trials with replication-competent oncolytic VACVs.

Strain	Virus name	Deletion gene	Insertion gene	Cancer type	Combination therapy	Phase	Status	NCT number
Wyeth	Pexa-Vec/JX594	TK	GM-CSF	Colorectal cancer	Durvalumab +Tremelimumab (anti-PD-L1 + anti-CTL-4 antibody)	I/II	Active, not recruiting	NCT03206073
				Metastatic/advanced solid tumor	Ipilimumab (anti-CTL-4 antibody)	I	Completed	NCT02977156
				Colorectal carcinoma	Irinotecan (chemotherapy)	I/II	Completed	NCT01394939
				Metastatic melanoma	ZKAB001 (Anti-PD-L1 antibody)	I/II	Recruiting	NCT04849260
				Renal cell carcinoma	Cemiplimab (anti-PD-1 antibody)	I/II	Active, not recruiting	NCT03294083
				Peritoneal carcinomatosis of ovarian cancer origin		II	Withdrawn	NCT02017678
				Hepatic carcinoma		I	Completed	NCT00629759
				Hepatocellular carcinoma		II	Completed	NCT00554372
				Hepatocellular carcinoma		II	Completed	NCT01387555
				Hepatocellular carinoma		II	Completed	NCT01636284
				Hepatocellular carcinoma	Sorafenib (chemotherapy)	II	Completed	NCT01171651
				Hepatocellular carcinoma	Sorafenib (chemotherapy)	III	Completed	NCT02562755
				Breast cancer, Soft-tissue sarcoma	Cyclophosphamide + Avelumab (chemotherapy + anti-PD-L1 antibody))	I/II	Recruiting	NCT02630368
				Melanoma, Lung cancer, Renal cell carcinoma, Squamous cell carcinoma of the head and neck		I	Completed	NCT00625456
				Metastatic/refractory colorectal carcinoma	Recombinant Vaccinia GM-CSF	I	Completed	NCT01380600
				Metastatic/refractory colorectal carcinoma		I	Completed	NCT01469611
				Neuroblastoma, Rhabdomyosarcoma, Lymphoma, Wilm’s Tumor, Ewing’s sarcoma		I	Completed	NCT01169584
				Melanoma		I/II	Completed	NCT00429312
Lister	GL-ONC1/Olvi-Vec	TK, F145L, HA	Luc-GFP, β-glucuronidase, β-galactosidase	Ovarian cancer, Peritoneal carcinomatosis, Fallopian tube cancer	Bevacizumab (anti-VEGF antibody)	I/II	Completed	NCT02759588
				Peritoneal carcinomatosis		I/II	Completed	NCT01443260
				Advanced solid tumors		I	Completed	NCT00794131
				Solid organ cancers		I	Terminated	NCT02714374
				Cancer of head and neck		I	Completed	NCT01584284
				Lung cancer		I	Active, not recruiting	NCT01766739
				Platinum-resistant ovarian cancer, Fallopian tube cancer, Primary peritoneal cancer, High-grade serous ovarian cancer, Endometrioid ovarian cancer, Ovarian clear cell carcinoma	carboplatin or cisplatin (chemotherapy), Bevacizumab (anti-VEGF antibody)	III	Recruiting	NCT05281471
	VV-GMCSF-Lact	TK, VGF	GM-CSF, Lact	Recurrent/refractory metastatic breast cancer		I	Recruiting	NCT05376527
	ASP9801	VGF, O1L	IL7, IL-12	Advanced/metastatic solid tumors	Pembrolizumab (anti-PD-1 antibody)	I	Active, not recruiting	NCT03954067
Copenhagen	TG6050	TK、RR	CTLA-4, IL-12	Non-small cell lung cancer		I	Recruiting	NCT05788926
	BT-001	TK、RR	CTLA-4, GM-CSF	Metastatic cancer, Soft tissue sarcoma, Merkel cell carcinoma, Melanoma, Triple negative breast cancer, Non-small cell lung cancer	Pembrolizumab (anti-PD-1 antibody)	I/II	Recruiting	NCT04725331
	TG6002/T601	TK、RR	FCU1	Glioblastoma	5-flucytosine (chemotherapy)	I	Unknown status	NCT03294486
				Advanced solid tumors	5-flucytosine (chemotherapy)	I/II	Unknown status	NCT04226066
WR	RGV004	TK	CD19BiFTE	Relapsed or refractory B-cell lymphoma		I	Recruiting	NCT04887025
	vvDD/JX-929	TK, VGF	CD/SMR	Melanoma, Breast cancer, Head and neck squamous cell cancer, Liver cancer, Colorectal cancer, Pancreatic adenocarcinoma		I	Completed	NCT00574977

## 7 Delivery route of oncolytic VACVs

Up to now, all the approved OVs are intratumorally injected, which renders them ineffective for cancers that are difficult for *in situ* administration or have been already metastatic. Developing intravenous OVs is essential to broaden the clinical applications of OVs. The data about 97 independent clinical trials reporting OV studies from 2000 to 2020 showed that the most common route was intratumoral delivery used in 48 of the clinical trials (49.5%) followed by intravenous delivery used in 34 of the clinical trials (35%) (121). In the clinical trials, intravenous oncolytic VACVs exhibit good safety and potential anti-tumor activity. Different from adenoviruses, oncolytic VACVs can be administrated once *via* intravenous injection because there are no preexisting neutralizing antibodies in most of human bodies. However, after oncolytic VACV treatment, neutralizing antibodies generation occurred, limiting the repeated systematic delivery.

Recently, researchers are dedicated to developing diverse approaches to realize the efficient tumor treatment by repeated administration of intravenous oncolytic VACVs. Ferguson et al. found that transient inhibition of PI3Kδ by the PI3Kδ-selective inhibitor IC87114 or the clinically approved idelalisib (CAL-101) prior to intravenous delivery of a tumor-tropic VACV could inhibit viral attachment to, but not internalization by, systemic macrophages through perturbation of signaling pathways involving RhoA/ROCK, AKT, and Rac, thus potentiating intravenous delivery of oncolytic VACVs to tumors (115). In addition, COX-2 inhibitor treatment can enhance the long-term protective anti-tumor effects generated by oncolytic VACVs *via* inhibiting the generation of neutralizing antibodies against oncolytic VACVs infection, enabling the repeated administration of oncolytic VACVs (116). Another study from McCart’s group demonstrated that CP40 (a complement inhibitor) pretreatment elicited an average 10-fold increase in infectious titer in the blood early after the JX-594 infusion by preventing oncolytic VACVs neutralization, beneficial for repeated intravenous administration of oncolytic VACVs (122). The expression of human CD55 protein by oncolytic VACVs could also prolonged viral survival by protecting against complement-mediated lysis and evading neutralization by VACV-specific antibodies, improving intravenous efficacy (123). All above approaches are theoretically and clinically feasible *via* antiviral inhibitors treatment prior to intravenous injection of oncolytic VACVs.

Besides inhibition of antiviral immunity, direct oncolytic VACVs shielding is another alternative to enhance the anti-tumor efficacy of intravenous oncolytic VACVs. As a non-enveloped virus, adenovirus has been delivered by many kinds of cell carriers (e.g., mesenchymal stem cells, erythrocytes), nanomaterials (e.g., liposomes, exosomes) or polymers (e.g., poly(ethylene glycol) (PEG), polyamidoamine) to improves the pharmacokinetics (124, 125). Hill et al. found that polymer (Chol-PEG(10K)-NHS) coating reduced the binding of neutralizing anti-VACV antibodies and oncolytic VACVs and increased the circulation time *in vivo*, although VACV is an enveloped virus (126). These results showed that VACV coating may be an effective way to improve the systematic delivery of oncolytic VACVs. Many other kinds of methods for VACV coating need to be developed.

## 8 Conclusions and prospects

With the continuous improvement of gene function and anti-tumor mechanism of oncolytic VACVs, a variety of genetically functional VACVs have been constructed and shown safety and efficacy in preclinical and clinical studies. In addition, VACV-based OVT has been acknowledged as a potential adjuvant immunotherapy when combining with traditional anti-tumor therapies. However, challenges remain in the clinical application of engineered oncolytic VACVs. Firstly, failure of the phase III trial about JX-594 reminds us the importance of selecting proper combination therapy, and ensuring the administration sequence and timing. In addition, the two sides of oncolytic VACV-mediated antiviral immunity should be overall considered during the construction of VACV backbones and gene engineering of oncolytic VACVs. Another challenge is repeated intravenous injection of oncolytic VACVs with lower dosages. It is acknowledged that natural tumor tropism and none of initial neutralizing antibodies make VACVs potential for a single intravenous injection, while repeated administration can activate strong antiviral immunity to eliminate VACVs, indicating that higher dosage is needed to achieve anti-tumor effect. With the discovery of diverse signal pathways that are related to antiviral immunity, some drugs show the positive effect on the VACVs circulation *in vivo*, facilitating the progress of VACVs systematic administration. While much work remains, lots of studies have shown the enormous potential of VACV-based OVT. We believe it will not be long before oncolytic VACVs are approved for clinical anti-tumor therapy.

## Author contributions

LX: Conceptualization, Writing – original draft. HS: Writing – review & editing. NL: Writing – review & editing. YX: Writing – review & editing, Supervision. PW: Supervision, Writing – review & editing, Conceptualization, Funding acquisition, Project administration.
